# Patient Work Personas of Type 2 Diabetes—A Data-Driven Approach to Persona Development and Validation

**DOI:** 10.3389/fdgth.2022.838651

**Published:** 2022-06-23

**Authors:** Natasha Galliford, Kathleen Yin, Ann Blandford, Joshua Jung, Annie Y. S. Lau

**Affiliations:** ^1^UCL Interaction Centre, University College London, London, United Kingdom; ^2^Centre for Health Informatics, Australian Institute of Health Innovation, Macquarie University, North Ryde, NSW, Australia

**Keywords:** patient work, type 2 diabetes, persona, persona validation, health informatics

## Abstract

**Introduction:**

Many have argued that a “one-size-fits-all” approach to designing digital health is not optimal and that personalisation is essential to achieve targeted outcomes. Yet, most digital health practitioners struggle to identify which design aspect require personalisation. Personas are commonly used to communicate patient needs in consumer-oriented digital health design, however there is often a lack of reproducible clarity on development process and few attempts to assess their accuracy against the targeted population. In this study, we present a transparent approach to designing and validating personas, as well as identifying aspects of “patient work,” defined as the combined total of work tasks required to manage one's health and the contextual factors influencing such tasks, that are sensitive to an individual's context and may require personalisation.

**Methods:**

A data-driven approach was used to develop and validate personas for people with Type 2 diabetes mellitus (T2DM), focusing on patient work. Eight different personas of T2DM patient work were constructed based physical activity, dietary control and contextual influences of 26 elderly Australian participants (median age = 72 years) *via* wearable camera footage, interviews, and self-reported diaries. These personas were validated for accuracy and perceived usefulness for design, both by the original participants and a younger (median age bracket = 45–54 years) independent online cohort f 131 T2DM patients from the United Kingdom and the United States.

**Results:**

Both the original participants and the independent online cohort reported the personas to be accurate representations of their patient work routines. For the independent online cohort, 74% (97/131) indicated personas stratified to their levels of exercise and diet control were similar to their patient work routines. Findings from both cohorts highlight aspects that may require personalisation include *daily routine, use of time*, and *social context*.

**Conclusion:**

Personas made for a specific purpose can be very accurate if developed from real-life data. Our personas retained their accuracy even when tested against an independent cohort, demonstrating their generalisability. Our data-driven approach clarified the often non-transparent process of persona development and validation, suggesting it is possible to systematically identify whether persona components are accurate or. and which aspects require more personalisation and tailoring.

## Introduction

Designing digital technologies to help people managing chronic health conditions requires a comprehensive understanding of patients' work and needs. Patient-centered health design uses a range of methods, such as interviews and observations, to achieve this goal (1, 2). Personas aim to represent user group demographics, motivations, and challenges (1, 3), and can help designers better understand patient work and needs (1). Many different personas types exist, such as those that focus on the ultimate goal of the user, focus on the role the user plays in the organization, or fictional personas based on the experience of the design team (4). Personas have been used to develop many digital tools supporting chronic condition self-management, and their applicability in Type 2 diabetes (T2DM) (5), heart disease (6), and multiple sclerosis (7) self-management has been explored.

In terms of health outcomes, personas contain great potential to improve the lives of T2DM patients. Incorporating quantitative data trends and qualitative context factors, personas can help to better implement health interventions for chronic diseases such as T2DM in sociotechnical contexts (8). In particular, self-management of complex, chronic conditions, such as T2DM, is related to a myriad of qualitative contexts such as culture, habit, routine and financial constraints, all of which impacts the efficacy of self-management often without being reported to the clinician (9). Personas highlight such influences and describes the reason behind patient behavior (1, 10), supporting designers, researchers, and clinicians to create more applicable intervention strategies for self-management. Behavioral interventions for chronic disease management designed with the help of personas have been shown to be effective in real patients (10, 11), indicating the benefit of personas in tailoring interventions for the T2DM patient group.

However, given the diverse nature of patient self-management, it is unclear how representative these health-related personas truly are. Personas can be abstract, impersonal, misleading, and distracting (12). Designers also often describing T2DM personal development methodologies vaguely and describes commonalities were created out of “general factors” (5), making the personas difficult to validate or verify (13). This makes it difficult to validate personas' accuracy, generalisability, and effectiveness for design. Validations of these personas in healthcare digital design are limited with only a handful of studies (11). Literature reviews showed ~30% of persona literature has been validated (14), either quantitatively [such as calculating Chi-squared tests for variables (15)] or qualitatively (using interviews), but with qualitative validation often being informal and not thoroughly described (14), undermining the strength of the validation process and presenting a gap in knowledge.

In this paper, we improve the persona design process by proposing a data-driven approach to developing and validating personas, focusing on the “patient work” of T2DM patients living in the community (16, 17). “Patient work” collectively refers to the large variety of tasks that people must conduct during self-management of health conditions, and the comprehensive contextual factors that alter how these tasks are performed (18, 19). Patient work takes place within an ergonomics system that is influenced by different levels of contextual factors, including contexts that came from the work itself (such as the person, the task itself, or the tools used) (9). Contextual factors outside of the work are split into physical, social, and organizational contextual factors, describing the physical environment, social circles, and temporal management, respectively (6, 9). We decided to incorporate the external contextual factors, as well as the tasks and routines of our patients, as the backbone of our persona development. We created health-focused personas that portrayed what the participant did at what time, how they did it, and why they did it. With such personas, we hope to provide insight on practical ways participants could be assisted in their self-management, and how they could be assisted more comprehensively through well-timed momentary interventions by digital devices or through psychosocial interventions such as offering a list of social security payments a poor family might be eligible for.

### Objective

In this paper, our goal is to make the persona development process more transparent, and generate accurate and relevant personas based on real patient data. We also aim to validate personas, both by getting feedback from the patients who contributed the data and by using a cohort in a different country and setting, thus understanding how representative these personas and specific components are for both cohorts. With this data, we can then pinpoint which components of personas would benefit from further personalized data, and which components are more generally applicable but do not generate as much impact on individuals.

## Methods

### Data Collection

This publication uses the data set collected from Yin et al. (17), which was previously published (16). Detailed data collection methodology and ethics permissions are provided elsewhere (17). Briefly, 26 participants with T2DM and at least one other chronic co-morbidity were recruited in public and private endocrinology specialist clinics in Sydney, Australia. They were interviewed regarding their self-management, health history, and daily routines, and were then given a wearable camera for 1 day (the waking hours of 1 entire day) to record their activities. Patients were also provided with a Time-Use Diary (20) to self-report their activities and to complement the camera recording. The Time-Use Diary asked participants to report, in 5-min blocks, their main activity, any concurrent activities, the participant's physical contexts, who the participant was with, and their level of enjoyment from the activity. The participants then conducted an exit survey after 24 h, where researchers downloaded the camera footage and reviewed it with participants, deleting any that the participant preferred to remove. The footage was then converted to 1 screenshot every 10 s using in-house code that automated the screenshot function of VLC media player (VideoLan, an open-source software) and the screenshots were manually analyzed. More details can be found in our protocol paper (17). This data collection was carried out by KY, JJ, and AYSL.

Table 1 outlines the demographic characteristics of the 26 original participants from whom the personas were constructed. This table is modified from our previous publication (16) and also includes the participants who attended our persona validation interviews.

**Table 1:** Original participant demographics (*n* = 26).

**Characteristics**	**Original participants (*n* = 26)**	**Persona validation participants (*n* = 10)**
**Gender**		
Male	16	7
Female	10	3
**Age**		
<60	2	0
60–64	3	0
65–69	3	2
70–74	6	4
75–79	7	2
80–84	2	0
85–89	3	2
**Insulin**		
Yes	16	5
No	10	5
**Number of years since T2DM diagnosis**		
<10 years	3	1
10–14 years	5	2
15–19 years	5	1
20–24 years	5	3
25–29 years	3	1
>29 years	5	2
**Ethnicity**		
Anglo Australian	14	4
Chinese	4	2
Indian	2	2
Italian	2	0
Trinidad and Tobago	1	0
UK migrant	1	1
Indonesian	1	1
Sri Lankan	1	0
**Number of co-morbidities**		
1	4	1
2	9	4
3	1	0
4	4	2
5	3	1
6–10	4	1
>10	1	1
**Employment**		
Retired	18	8
Self-employed	3	1
Employed by others	5	1

### Persona Development

The researchers NG, AB, and AYSL conducted the following section of work. A case profile was developed for each of the 26 participants, capturing all the participants' self-management behaviors and contextual influences. All quantitative and qualitative data sources mentioned in previous publications (camera screenshots, interview transcripts, Time-Use Diary, researchers' field notes, photos of the participant's dwelling, etc.) were used to develop these case profiles (16, 17). We utilized a within-case studies approach (21), identifying the unique attributes and contextual experiences of each case profile before identifying general patterns across participants. Specifically for persona development, we established four components in our personas, based on the different data we obtained from various instruments during the study and the information presented in the case profiles.

*1) Quotes and Summary*—This component includes a representative quote describing the persona's patient work, followed by a prose summary of the persona's self-management behavior pattern. This information is collected from interview transcripts, photos, and researchers' field notes of all participants belonging to that persona.
*2) Time-spent Bar Graphs*—This component includes bar graphs that quantitatively demonstrate, on average, how many minutes participants in that persona spent doing specific self-management activities per day (total number of minutes per task per persona divided by number of participants contributing to the persona). The activities tracked here are medication (including managing and taking medication), exercise, food (including preparing and ingesting food), and using portable electronic devices (such as phones and tablets). This information is collected from participants' wearable camera screenshots, which are timestamped, and the Time-Use Diary, which is recorded in 5-min intervals.
*3) Contextual Influences*—This component includes all physical, social, and organizational contextual factors which have affected the self-management routines of participants belonging to that persona. Information regarding physical contexts is obtained from researcher's photos, field notes, and camera screenshots. Information regarding social and organizational contextual factors is obtained from researcher's field notes and interview transcripts.
*4) Activity Timeline*—This component represents a persona's daily routines that incorporates temporal data of all participants belonging to that persona in visual timeline form, indicating what these participants did at different times of the day and for how long, and their mood during that task. This information is obtained from the Time-Use Diary and the wearable camera screenshots.

In total, eight personas were formed by grouping the 26 participants' case profiles according to three factors: self-reported levels of physical activity, diet control, and influence of contextual factors in managing T2DM (internal vs. external). These three factors were selected to define personas because the first two (physical activity, diet control) are important behaviors that can be encouraged to change to support good control of T2DM. Physical activity and diet control were also observed as two main axes upon which the data could be divided, with some participants clearly very concerned about diet (or exercise) and others caring very little about diet (or exercise), with no participant falling into the middle ground.

The last factor (internal influence e.g., self vs. external influence e.g., social) was selected because one's locus of control (i.e., the degree to which people believe that they, as opposed to external forces, have control over the outcome of events in their lives) would have a significant impact on how designers and researchers should approach behavioral change intervention (22–24). In this study, we did not use demographic factors such as gender, nationality to define our personas because we want to focus on factors that participants have the means to change (e.g., T2DM-related behaviors, locus of control). Furthermore, our data was not collected to study how specific demographic factors affect T2DM-related behaviors.

Overall, personas were classified as having (i) high/moderate physical activity vs. less physical activity, (ii) high/moderate diet control vs. less diet control, and (iii) mostly influenced by internal factors within the self (e.g., contextual factors resulted in altered motivation, prioritization, etc.) or external factors from other people (e.g., contextual factors from family and friends).

Participants were considered to have high or moderate physical activity if they met two or more of the following criteria:

1) Engage in deliberate exercise of more than 90 min per week,
2) Monitor the amount of physical activity they complete through tracking progress,
3) Incorporate exercise into their weekly activities *via* a routine,
4) Receive specialized exercise support or attend a gym.

Participants were considered to have high or moderate diet control if they met three or more of the following criteria:

1) Only eat foods with high sugar content in moderation,
2) Reduce portion sizes or remove food groups to meet weight loss goals,
3) Track diet or weight loss,
4) Rarely eat out of the home for social reasons or convenience (once a week at most),
5) Rarely opt for quick, fast, or frozen foods (once a week at most).

All information above were able to be collected quantitatively due to our mixed-methods methodology of time-stamped video footage, self-reported Time-Use Diary, and interviews. The video footage and diaries allowed us to derive data on how many minutes each person spent on specific work on the day of the study, and interview data allowed us to identify how frequently participant did specific tasks (such as eating out).

Participants were considered to be more likely to be under social influence if they reported more external factors (e.g., friends, families) than internal factors (e.g., motivation, prioritization) when reporting factors that influence their T2DM-related behaviors, such as diet and physical activity.

Table 2 depicts persona number and participant IDs categorized in that persona group. All eight personas can be found in Appendix 1.

**Table 2:** Persona characteristics.

**Persona number**	**Physical activity**	**Diet control**	**Contextual influence**	**Participant IDs**
1	High / Moderate	High / Moderate	Self	3, 4, 14
2	High / Moderate	High / Moderate	Social	1, 2, 17, 18, 20
3	High / Moderate	Low	Self	15, 19, 26
4	High / Moderate	Low	Social	9, 10, 21
5	Low	High / Moderate	Self	7, 13
6	Low	High / Moderate	Social	8, 11, 22
7	Low	Low	Self	6, 16, 23
8	Low	Low	Social	5, 12, 24, 25

### Persona Feedback From Original Participants

The persona profiles were taken back to the original participants in Sydney, Australia, for within-case validation and feedback (21) by the researchers KY and JJ. The participants were invited to a 1-h face-to-face semi-structured interview where they viewed a printed version of the persona they fitted into. Of the 26 original participants, 10 agreed to the interview. The participants who agreed to interview are P01, P03, P05, P06, P18, P19, P20, P22, P23, and P24, and their interview quotes are labeled as accordingly. They represent persona 1, 2, 3, 7, and 8. Participants were asked to “talk out loud” about their thoughts as they looked at the persona. The interviews were recorded and transcribed. They were asked to rate the four components of the persona (*Quotes and Summary, Time-spent Bar Graphs, Contextual Influences*, and *Activity Timeline*) from 1 (highest rank) to 4 (lowest rank) for perceived accuracy to themselves and usefulness to designing something to help them. They were then asked why they ranked each section at their respective positions. We allowed participants to rank multiple components in the same rank if they did not feel a difference. The interview question prompts can be found in Appendix 2.

The interview transcripts were analyzed in NVivo using thematic analysis (25). KY and JJ conducted all interviews and analyzed all transcripts. The two researchers read all transcripts, extracted codes from participants' answers, congregated them into themes, sorted themes and combined them, and finalized the theme table. Disagreements were resolved by consensus.

### Persona Feedback by Online Questionnaire From Other T2DM Patients

To assess whether these personas are indeed accurate representations of T2DM patient work behaviors, an independent cohort of people with T2DM (who were not involved in the data collection nor the persona development process) was recruited to provide feedback on these personas *via* a 10-min online questionnaire hosted on Qualtrics. This work was carried out by researchers NG and AYSL. A copy of the questionnaire is found in Appendix 3. Participants (*n* = 131) were gathered on Prolific (www.prolific.co), an online participant pool with participants from the United Kingdom and the United States for surveys, where each participant is paid a small fee. For this study, each participant received £1.40. To be eligible to answer the questionnaire, participants must be over 18 years old, have been diagnosed with T2DM, and fluent in English. Table 3 outlines the general demographics of these online survey participants.

**Table 3:** Online participant demographics (*n* = 131).

**Characteristics**	**Number of participants**
**Gender**	
Male	72 (55.0%)
Female	56 (42.7%)
No response	3 (2.3%)
**Age**	
18–24	1 (0.8%)
25–34	20 (15.3%)
35–44	27 (20.6%)
45–54	39 (29.8%)
55–64	32 (24.4%)
Older than 65	12 (9.2%)
**Insulin**	
Yes	34 (26.0%)
No	97 (74.0%)

Participants were stratified using two T2DM management questions. Participants were asked to rate their engagement in physical activity on a 6-point Likert scale from 1 (very low) to 6 (very high) and repeated for diet control. Ratings of 1–3 on the scale were considered “low,” and 4–6 considered “high.” We are unable to obtain precise data such as objectively recorded data and in-depth interviews from the online survey participants, and thus cannot apply our persona criteria. Instead, we opted to let the online participants self-evaluate their exercise and diet control. Participants were also asked what contextual factors in their lifestyle have a positive and negative impact on their management of T2DM.

Participants rating themselves as high or low in physical activity and diet control would view personas who had similar levels of physical activity or diet control. Each participant was shown the two personas that matched their physical activity or diet control, with the only difference between the two being the main source of contextual influences. Participants were provided with an explanation of what a persona was, asked to read each persona and to provide a rating of how similar the persona is to their T2DM self-management on a scale from 1 (very different) to 7 (very similar). They were then asked which component of the persona contains information that is similar and or different from their own experiences. Participants were provided with a list of components in the personas and asked to select all components that are similar or different to their lives, and did not use a ranking or a scale system (see Appendix 3).

## Results

### Persona Validation by Original Participants

**Figure 1:**
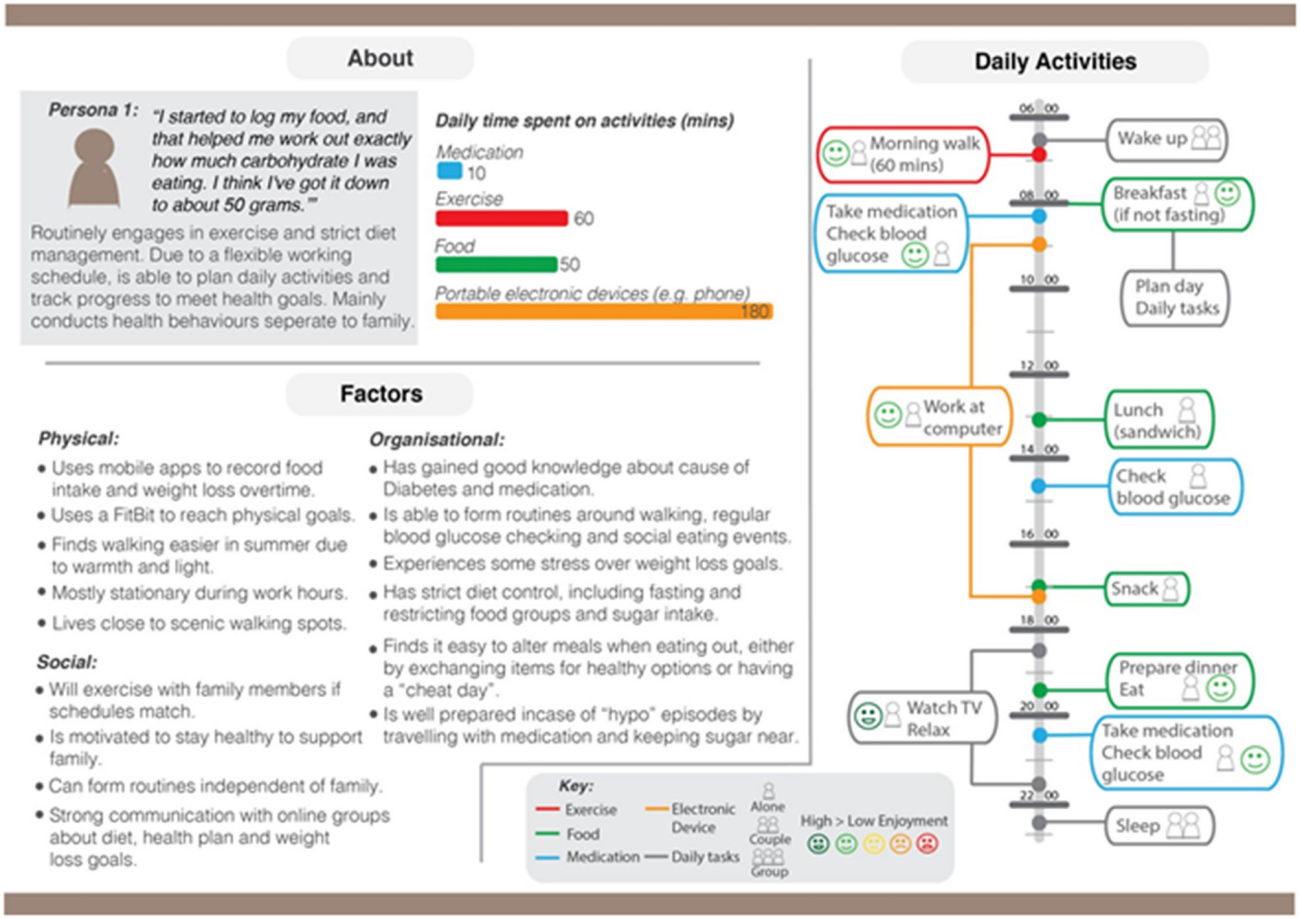
An example persona (high exercise, high diet control, and self-contextual factors).

Figure 1 illustrates an example of our personas. Each original participant only viewed the persona that their case profile fitted into.

In terms of perceived accuracy toward the participant's self-management (where ranking 1 was considered most accurate out of the four components; and four was least accurate of the four), the component considered most accurate was *Contextual Influences* (mean = 1.8), followed by *Quotes and Summary* (mean = 2.2) and *Activities Timeline* (mean = 2.2), with *Time-spent Bar Graphs* being considered the least accurate (mean = 3.3) of these four components.

In terms of perceived usefulness in designing digital interventions to help them (where ranking 1 = considered most useful out of the four components; and 4 = least useful of the four), the component considered most useful was *Activities Timeline* (mean = 1.8), followed by *Contextual Influences* (mean = 2.2), then *Quotes and Summary* (mean = 2.8), with *Time-spent Bar Graphs* at the lowest (mean = 3.3). For detailed bar-graphs, please refer to Appendix 4.

Overall, participants from the original cohort ranked *Contextual Influences* as the most accurate and second-most useful; *Activities Timeline* component as the most useful and second-most accurate; *Quotes & Summary* not as useful nor accurate (ranking second for accuracy and third for usefulness); and *Time-spent Bar Graphs* considered the least accurate and least useful, suggesting low acceptability and reliability for this feature.

For qualitative feedback, participants outlined the main reasons they would consider a persona (and its components) as accurate or useful. Participant feedback was collated into two themes: (1) Lack of variation improves accuracy, and (2) Ease-of-use underlies usefulness.

### Theme One: Lack of Variation Improves Accuracy

When asked why they considered certain components as more accurate than others, participants stated they generally attributed better accuracy for components that are objectively factual and concise. Any information that is unlikely to change on a long-term basis, such as *Contextual Factors*, are also considered more accurate compared to components that could change daily, such as *Time-spent Bar Graphs*. Participants discussed that more accurate information allows the viewer to perceive patterns of behavior, which would help to better manage health. The accuracy of the component was considered very valuable to participants, as higher accuracy was considered to show the level of organization one had in one's life.

“*I like the contextual factors… Factors, yeah. Because again it is quite concise and accurate”* (P1)

“*It just serves a regular pattern of life that I think assists in your control of Diabetes. So, that, to me, is the most important thing.”* (P20).

### Theme Two: Ease-of-Use Contributes to Usefulness

When asked about why they ranked some components as more useful to design support applications for them than others, participants revealed they considered ease-of-use as a significant contributor to perceived usefulness. Those included the level of clarity in the language used for that component of the persona, clarity in visual presentation, how easy it is to read, and how much detail is present. Participants valued how the *Activities Timelines* allows a large amount of information to be easily obtained and processed at one glance, “all clear in front of you,” “everything in your face,” as opposed to the other three components, which were considered “wordy” and “difficult to understand.”

“*Because it just is sort of like you can see it, everything in your face.”* (P23)

“*Timeline is just clear to see”* (P19)

“*I think that's very clear and very easy to read and understand.”* (P20).

### Persona Validation by Questionnaire

The anonymous survey conducted online showed that the personas were also found to be similar with other T2DM patients in the community.

All participants were shown two personas with similar levels of exercise and diet control to their self-reported levels, and asked to validate whether these two personas were similar to the participant's own self-management routines (on a 7-point Likert scale, where 1 = Very Different, 2 = Moderately Different, 3 = Somewhat Different, 4 = Neutral, 5 = Somewhat similar, 6 = Moderately similar, 7 = Very similar). Based on these ratings, out of the two personas every participant was asked to rank, 74% (97/131) of participants identified at least one persona that resembled their own self-management (i.e., a rating of “Somewhat similar” or higher), and approximately one third of participants (33.6%, 44/131) found at least one persona that was “Moderately similar” or “Very similar” to themselves.

Sub-group analyses were conducted to investigate which components of the personas were deemed similar or different. In total, each participant provided two responses to the questions relating to the two personas (i.e., two responses for two personas viewed), thus *n* = 262 responses (i.e., 131 × 2). To find out specifically which component in a persona may be sensitive to an individual's circumstances (and thus may benefit from personalisation), we asked each participant to identify all six components in a persona (*Quotes and Summary, Time-spent Bar Graphs, Activities Timeline, Social Contextual Influences, Physical Contextual Influences, Organizational Contextual Influences*) that were considered similar (or different) to them.

For those responses that rated a persona as similar (i.e., ratings of 5 or higher), the *Activity Timeline* (62.5%) was most frequently selected as a similar component to the participant's own management. *Social Contextual Influences* (46.6%) and *Time-spent Bar Graphs* (42.7%) were also frequently selected. Similarly, for responses that rated a persona as different to their own management, the *Activity Timeline* (58.1%) was most frequently selected as a feature different from the participant. Again, *Social Contextual Influences* (50.4%) and *Time-spent Bar Graphs* (49.6%) were features frequently identified as being different. See Table 4 for a full list of features selected as similar and selected as different.

**Table 4:** Persona components considered as similar or different.

**Persona components**	**Number of participants (*n* = 131)**
**Chosen as similar**	
Activity timeline	82/131 (62.5%)
Social contextual influences	61/131 (46.6%)
Time-spent bar graphs	56/131 (42.7%)
Physical contextual influences	53/131 (40.5%)
Organizational contextual influences	46/131 (35.1%)
Quotes and summary	42/131 (32.1%)
Other	4/131 (3.1%)
None	1/131 (0.8%)
**Chosen as different**	
Activity timeline	75/131 (57.3%)
Social contextual influences	65/131 (49.6%)
Time-spent bar graphs	64/131 (48.9%)
Physical contextual influences	62/131 (47.3%)
Organizational contextual influences	60/131 (46.6%)
Quotes and summary	55/131 (42.0%)
Other	5/131 (3.8%)
None	3/131 (2.3%)

## Discussion

Developing personas to understand how specific user subpopulations behave has been a long-standing practice in digital health design (1), however their level of accuracy to the intended subpopulation had not been commonly assessed (26). In this study, we developed eight personas on T2DM self-management based on real-life patient work data. We then took the personas to be validated both from the original Australian participants, and from an independent sample of the general T2DM population in the United Kingdom and United States. We discovered that the original participants considered the persona components *Contextual Influences* and *Quotes and Summary* to be more accurate, as these components contain information that is less likely to change on a daily basis. These participants considered *Activity Timeline* and *Contextual Influences* as most useful for designing interventions to fit their lives, due to the clearer and easy-to-read visual representation that communicates the information succinctly. Meanwhile, 74% of the online general T2DM sample considered their allocated personas to be accurate to their daily self-management, and considered *Activity Timeline, Social Contextual Factors*, and *Time-spent Bar Graphs* as both most similar and most different.

These findings reflect that some persona information would vary more between individuals and some vary less, offering opportunities to examine whether some persona information might be more useful for ecological momentary self-management interventions (27, 28). Components which were considered by the original participants to be more accurate included more generalized and unchanging information (e.g., contexts, life attitudes), whereas more individualized and variable information (e.g., daily routines, number of minutes spent on each activity every day) were considered by original participants as more likely to shift according to circumstances, and thus “less accurate.” This was echoed by findings in the online cohort, where these more varied and personalized information were ranked as both most similar and most different to individual participants.

Our data-driven personas retained their accuracy in the original and online cohorts, despite the age and nationality differences in circumstances between the two cohorts. The original participants involved in the persona development were on average older individuals, retired, Australian, and with advanced T2DM. The online cohort were younger, working adults living in the United Kingdom and United States and were at various stages of their T2DM status. Indeed, while T2DM is a multi-faceted systematic chronic disease, there are fundamental T2DM issues (such as diet control, exercise, and glucose management) shared across all T2DM patients (29). With the data-driven process, we were able to discern patterns in our participant data and stratify the cohort into 8 different personas, which allowed us to better target subpopulations of real T2DM patients and better reflect their individualized patient work. As mentioned previously, persona validation is not commonly carried out or recorded in literature (14). T2DM personas that are validated have played important roles in improving T2DM management, such as improving the use and acceptance of a diabetes self-management system in the community (30) or improving motivation and self-management behavior using learning paths tailored for personas (11), suggesting such personas have a role to play in contributing to better T2DM outcomes.

Therefore, it would be important for designers to decide what kind of information they wish to include for the issues they are designing personas for, such as including personality traits to anticipates patterns of use (31). While personas are commonly acknowledged to produce benefits such as being decision guides or help validate the final product (32), there are no guidelines on the extent of personalisation (such as personal routines, personality traits), with conflicting data on whether it is beneficial to have more “personal details” in personas (31, 32). Would it be more prudent to design persona components that are more generalized (e.g., Personal quote and summary, *Organizational Contextual Influences*) to appeal to a broader audience, but lose out on opportunities for tailored and potentially more useful designs that has a greater impact for relevant individuals? This is a conflict that most designers struggle with—finding the balance between designing for the majority vs. personalizing for the individual. By using a data-driven approach to validate personas, we have demonstrated it is possible to utilize real patient feedback to identify aspects of a persona that may require personalisation as they are sensitive to an individual's contexts (e.g., one's daily routine, use of time, and social context), and aspects that may be open to interpretation as they are not as sensitive to individual factors (e.g., physical and organizational contexts).

Our study has the fundamental strength of carrying out persona validation after making the personas, a step commonly ignored in studies reporting persona construction. Moreover, we conducted two phases of validation, one with original participants and one with a general sample not involved with persona development. This allowed us to examine both the accuracy of the persona construction process (by checking with the original participants) and the generalisability and accuracy in relation to the general targeted group (by checking with the online cohort), doubly ensuring the relevance of our personas. At the same time, this study does have some limitations. For example, the rules used to categorize participants based on levels of exercise and diet control have not been verified and do not necessarily imply optimal or suboptimal self-management choices. Rather, they were data-driven, emerging from the data as prevalent trends for participants involved in the study. Similarly, most of the data used to inform these persona choices was qualitative and open to interpretation. Nevertheless, the validation stage of this study suggests that the personas were relatable for most of the target user group, acting as an accurate representation for their intended patient subpopulation. We were also only able to arrange for 10 of the original 26 participants to conduct the validation interviews as some participants were not available at the time, and these 10 participants had only represented five out of the eight personas.

## Conclusion

This study had shown that data-driven persona development can retain high levels of accuracy for both participants who had contributed data to the personas and a general sample from a different nationality and age bracket. Stratifying users by existing behavior patterns, in this case with exercise and diet control for T2DM patients, contributes positively to perceived accuracy. Including persona components that contain more personalized and varied data, such as personal daily routines, run the risk of reduced accuracy with some participants but can increase accuracy with other participants, and have increased potential for more individually impactful interventions or nudges. Digital health designers looking to develop more targeted interventions could perhaps improve the accuracy of such personas by including such information. On the other hand, persona components that are more generalized, such as a prose personality summary, are accepted as relatively accurate by a larger number of people but do not speak for anyone in particular. Designers looking to design interventions that act on a population-scale, or to improve general knowledge, might benefit more from including such information in their personas.

## Data Availability Statement

The raw data supporting the conclusions of this article will be made available by the authors, without undue reservation.

## Ethics Statement

The studies involving human participants were reviewed and approved by Macquarie University Human Research Ethics Committee for Medical Sciences (reference number 5201700718). University College London Research Ethics Committee (reference number UCLIC/1819/006/BlandfordProgrammeEthics). The patients/ participants provided their written informed consent to participate in this study. Written informed consent was obtained from the individual(s) for the publication of any potentially identifiable images or data included in this article.

## Author Contributions

NG constructed the personas, conducted the online validation study, and analyzed the data from the online study. KY and JJ conducted the original participant face-to-face interviews and analyzed the data from the interviews. KY and NG wrote the first draft of the manuscript. AB and AL conceptualized the studies and provided feedback on the manuscript. All authors contributed to the article and approved the submitted version.

## Funding

AL was supported by the New South Wales Health Early-to-Mid Career Fellowship. KY was supported by the National Health and Medical Research Council (NHMRC) Center for Research Excellence in Digital Health (APP1134919).

## Conflict of Interest

The authors declare that the research was conducted in the absence of any commercial or financial relationships that could be construed as a potential conflict of interest.

## Publisher's Note

All claims expressed in this article are solely those of the authors and do not necessarily represent those of their affiliated organizations, or those of the publisher, the editors and the reviewers. Any product that may be evaluated in this article, or claim that may be made by its manufacturer, is not guaranteed or endorsed by the publisher.
