# Supplementary material for: Patient Work Personas of Type 2 Diabetes—A Data-Driven Approach to Persona Development and Validation
Source: Front Digit Health. 2022 Jun 23;4:838651. doi: 10.3389/fdgth.2022.838651 (PMC9260172; doi:10.3389/fdgth.2022.838651)

Appendix 1 – The eight personas formed in the study

Persona 1: High/moderate exercise engagement and diet control. Mainly influenced by self- motivation and independence.

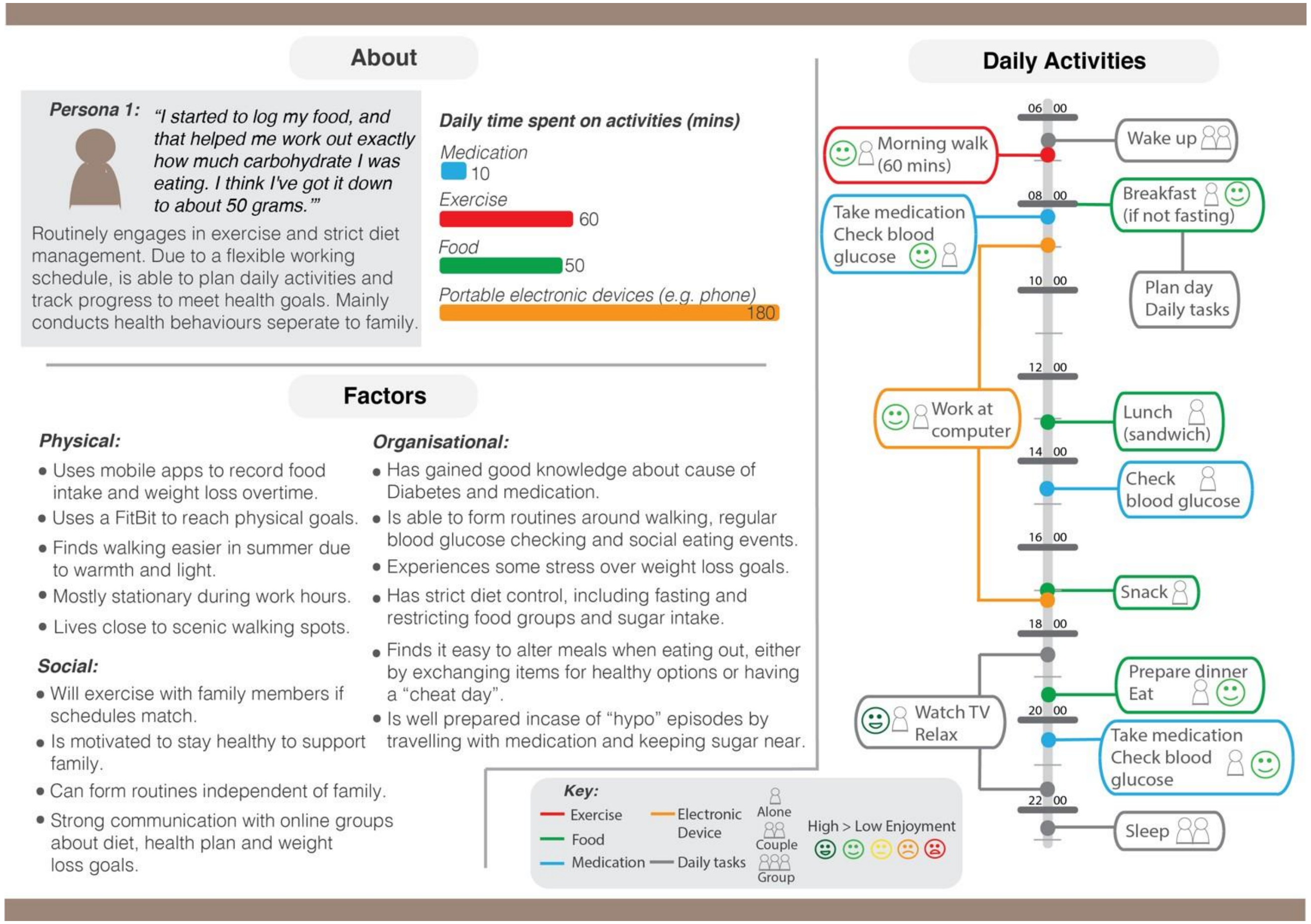

Persona 2: High/moderate exercise engagement and diet control. Mainly influenced by family.

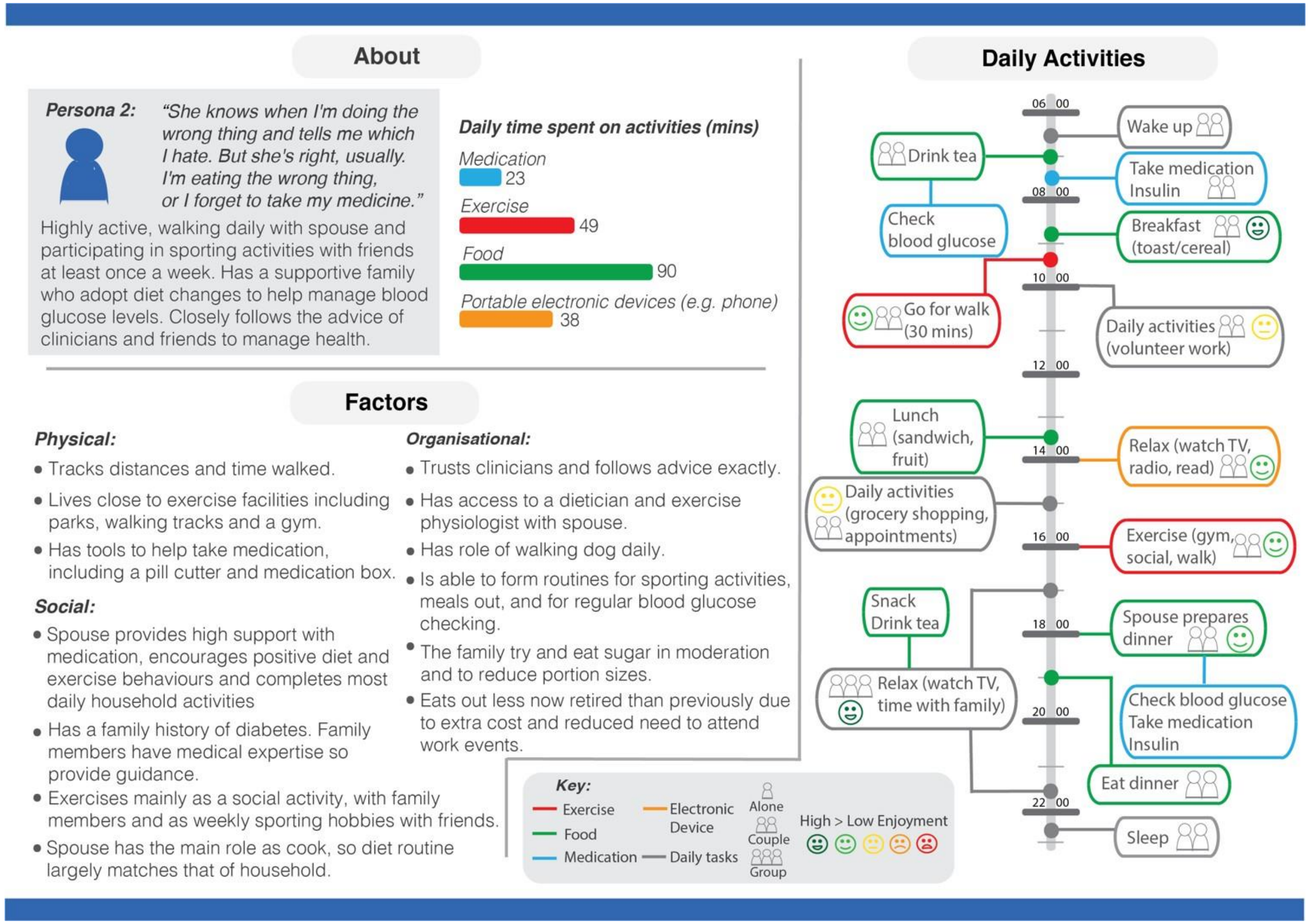

Persona 3: High/moderate exercise engagement. Low diet control. Mainly influenced by employment factors (such as log work hours) and energy levels.

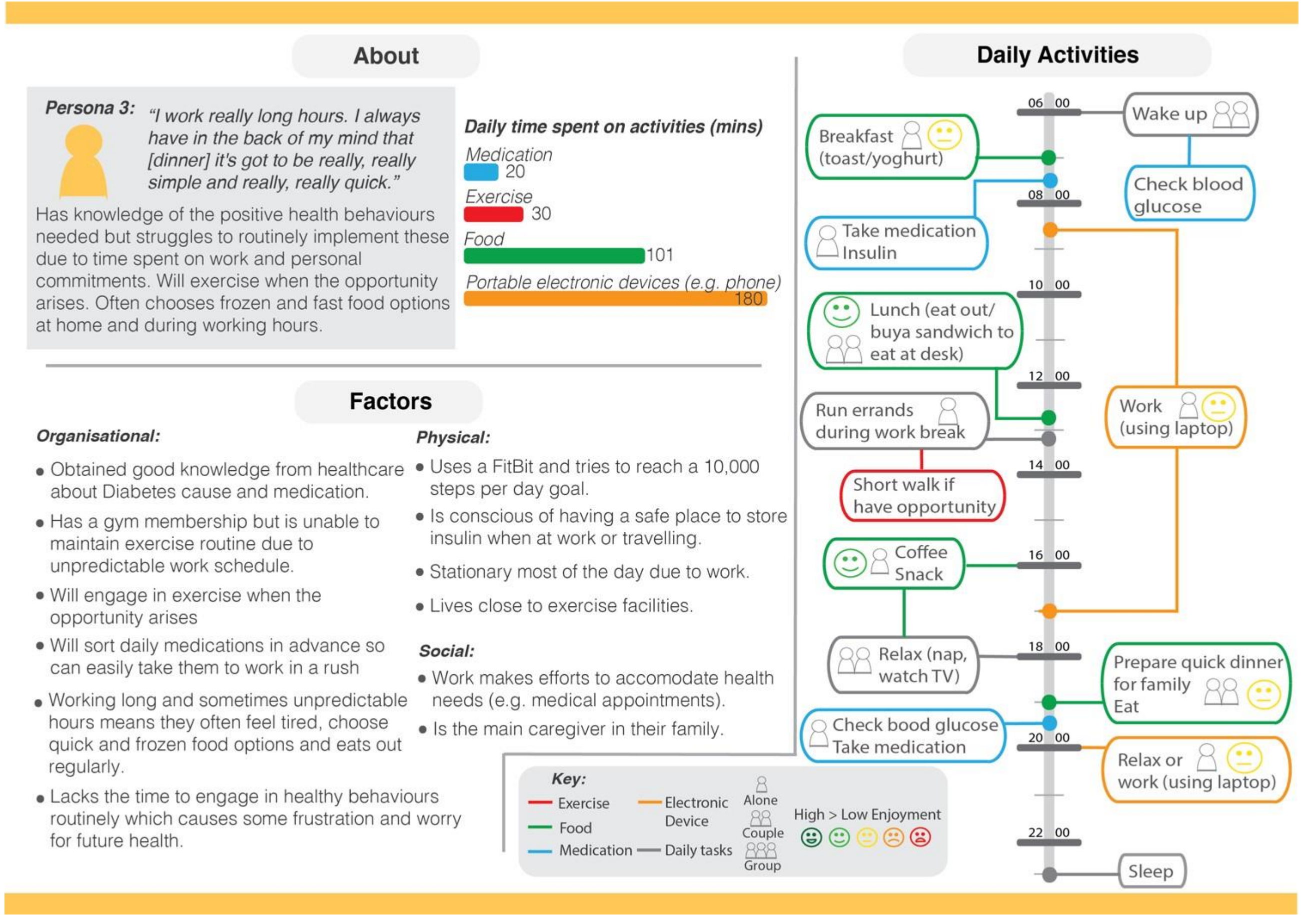

Persona 4: High/moderate exercise engagement. Low diet control. Mainly influenced by social group activities.

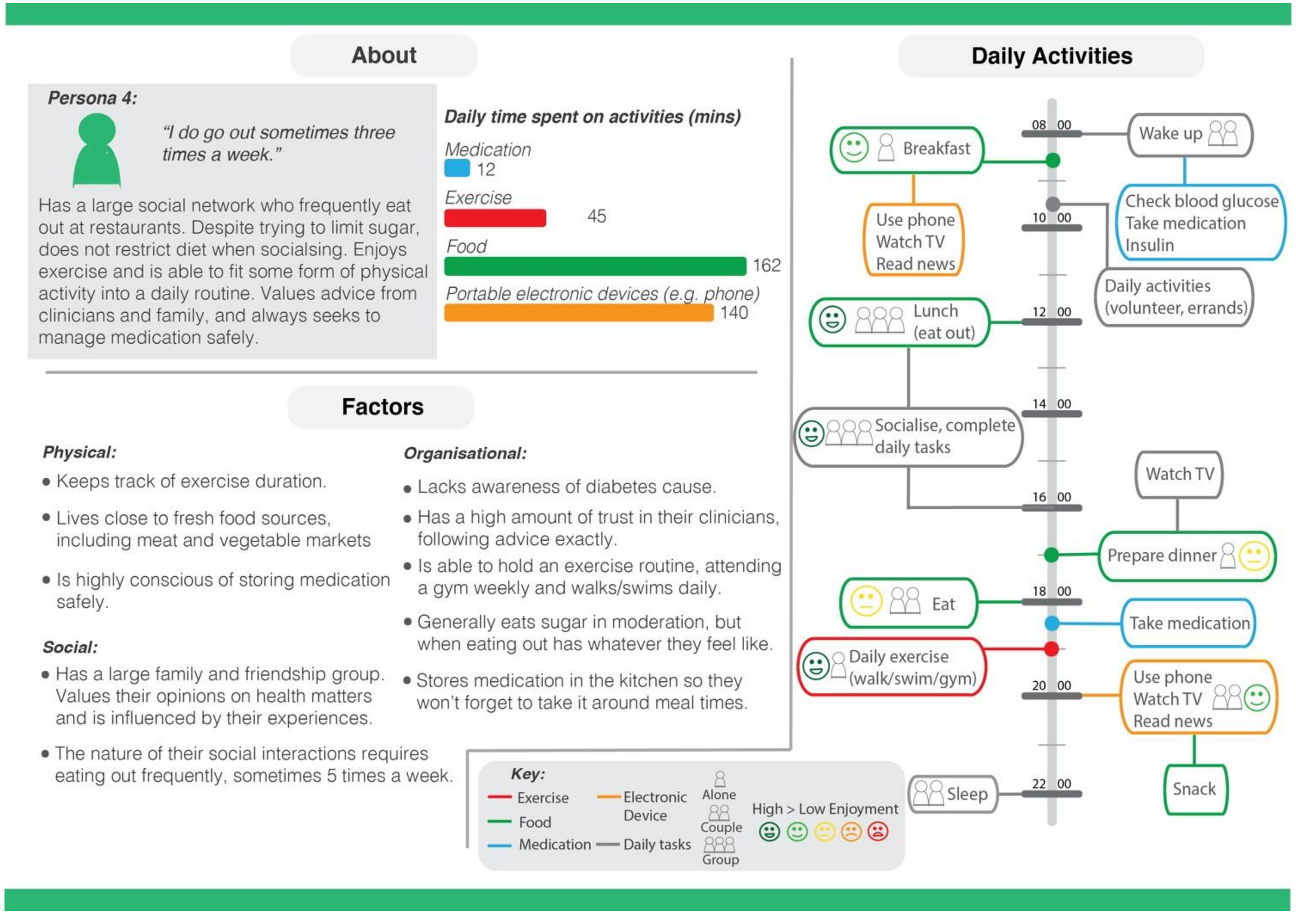

Persona 5: Low exercise engagement. High diet control. Mainly influenced by stressors and competing responsibilities in daily life.

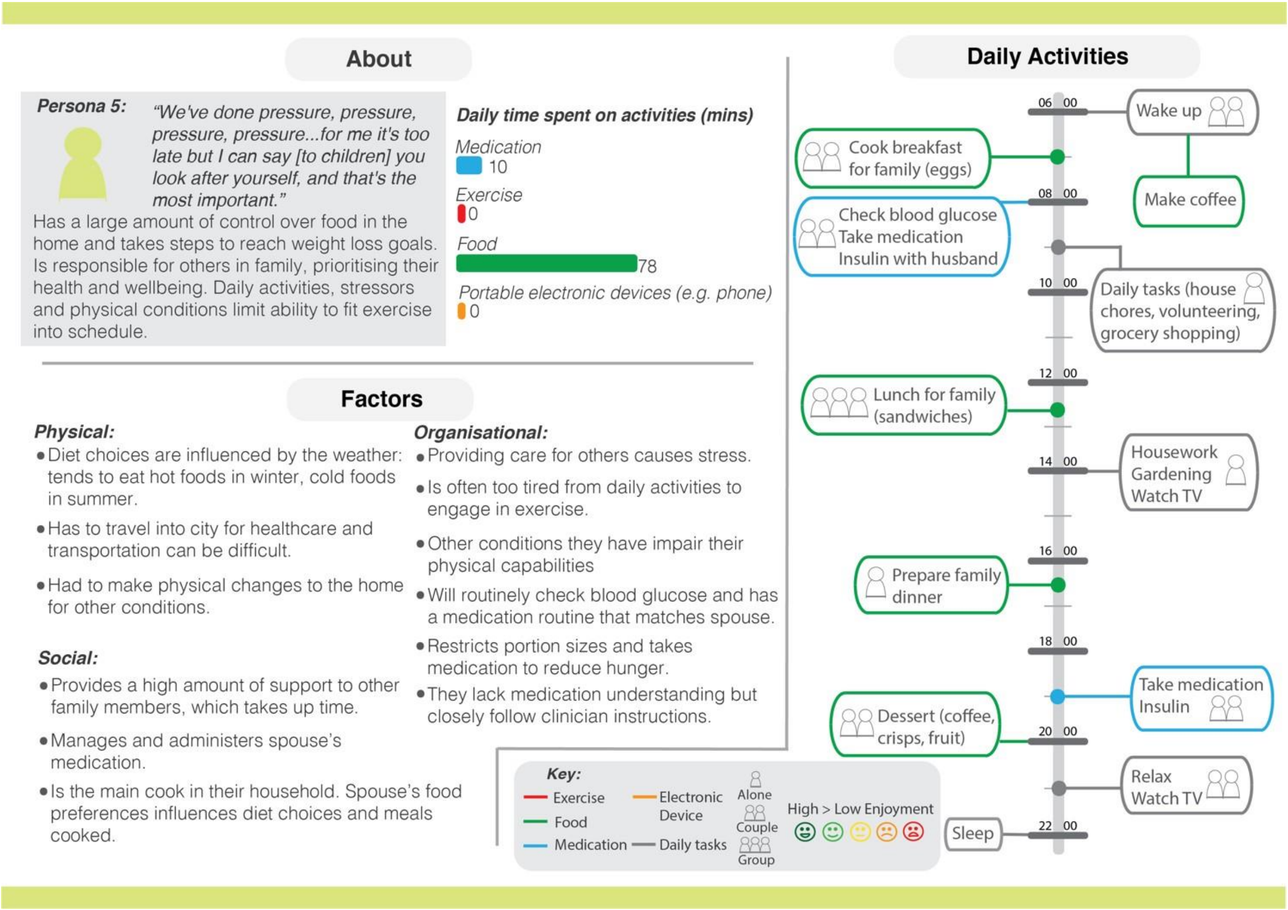

Persona 6: Low exercise engagement. High diet control. Mainly influenced by high support from others but lacks self-motivation.

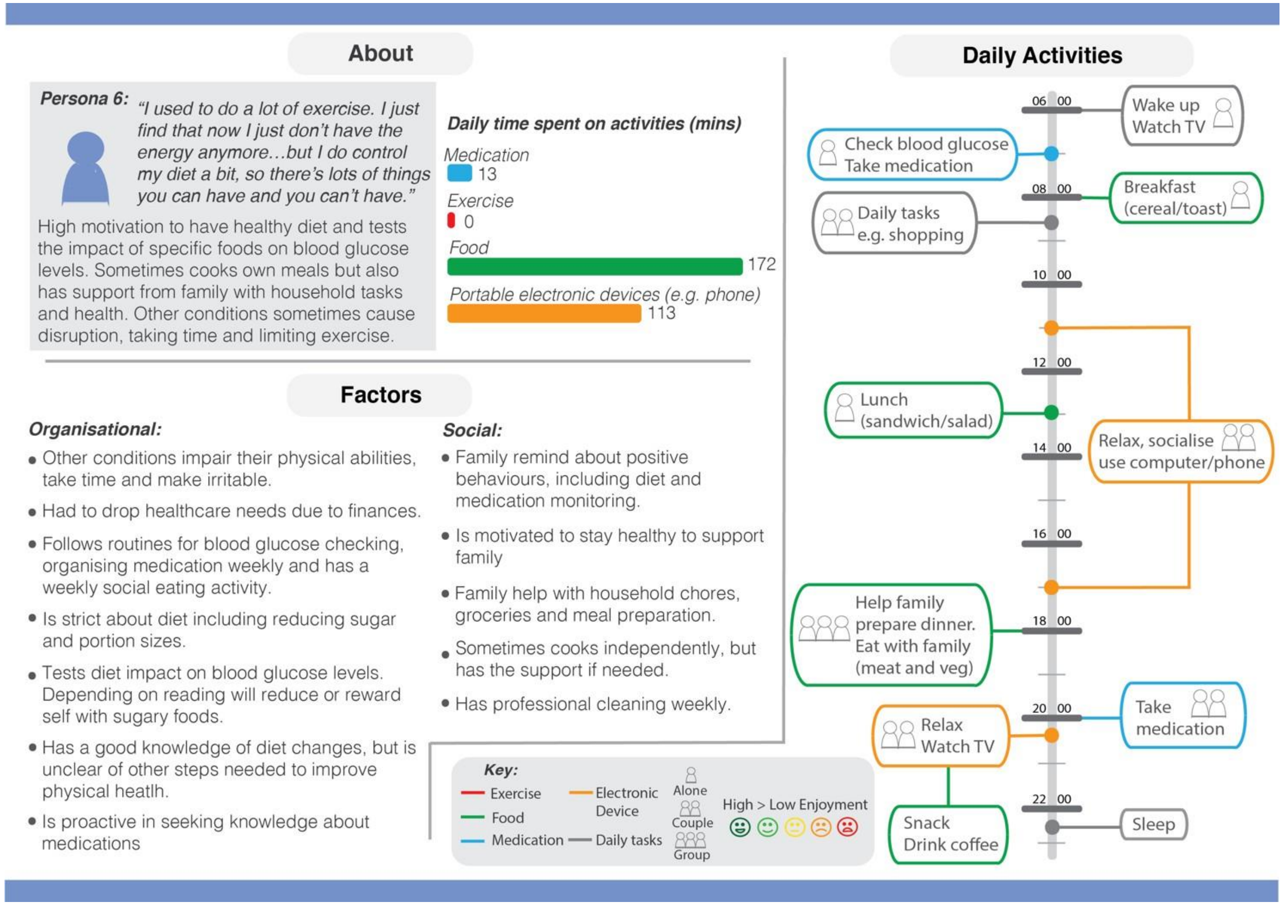

Persona 7: Low exercise engagement. Low diet control. Lacks motivation and support and prioritises other life priorities.

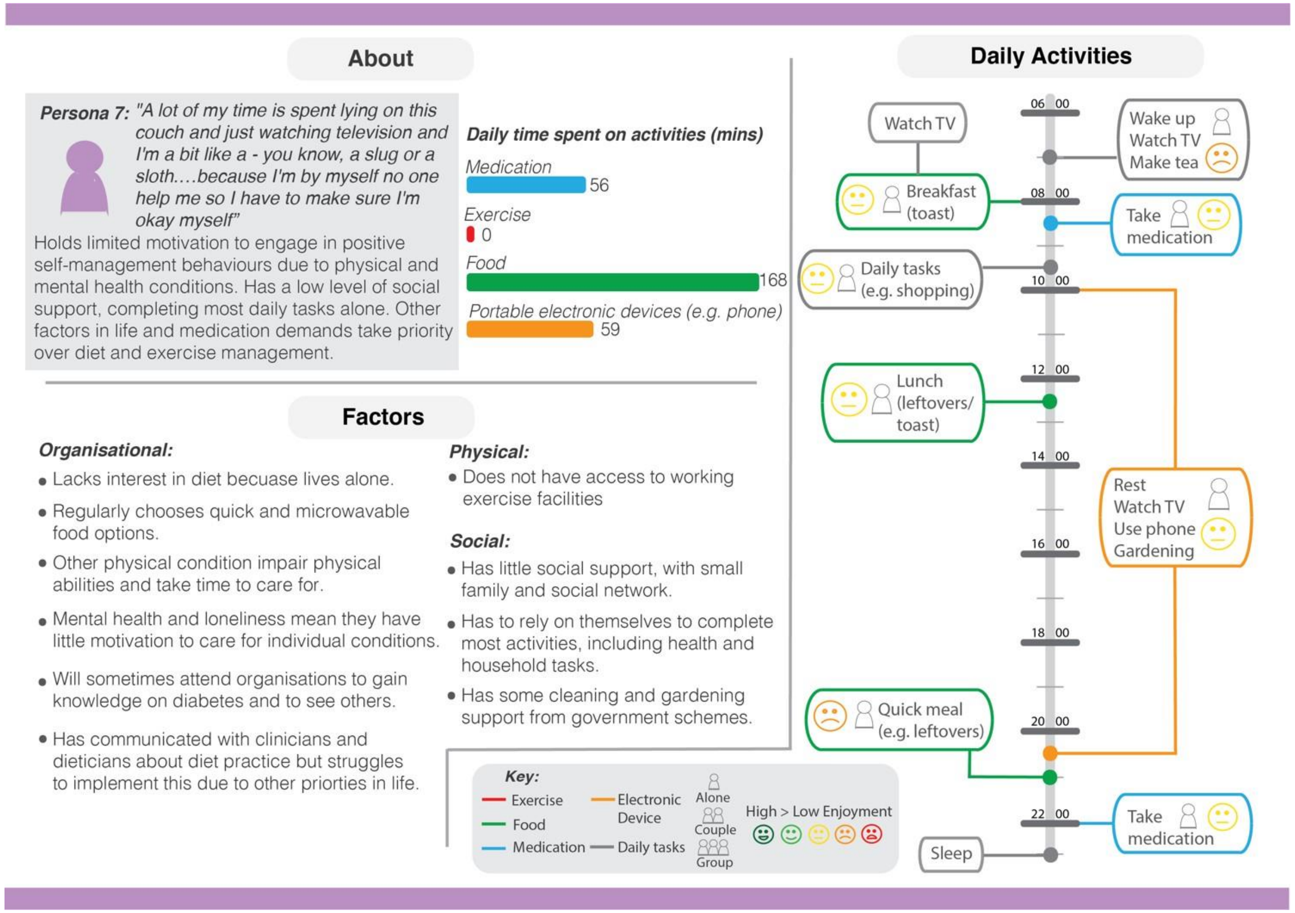

Persona 8: Low exercise engagement. Low diet control. Mainly influenced byfamily influence but has low self-motivation.

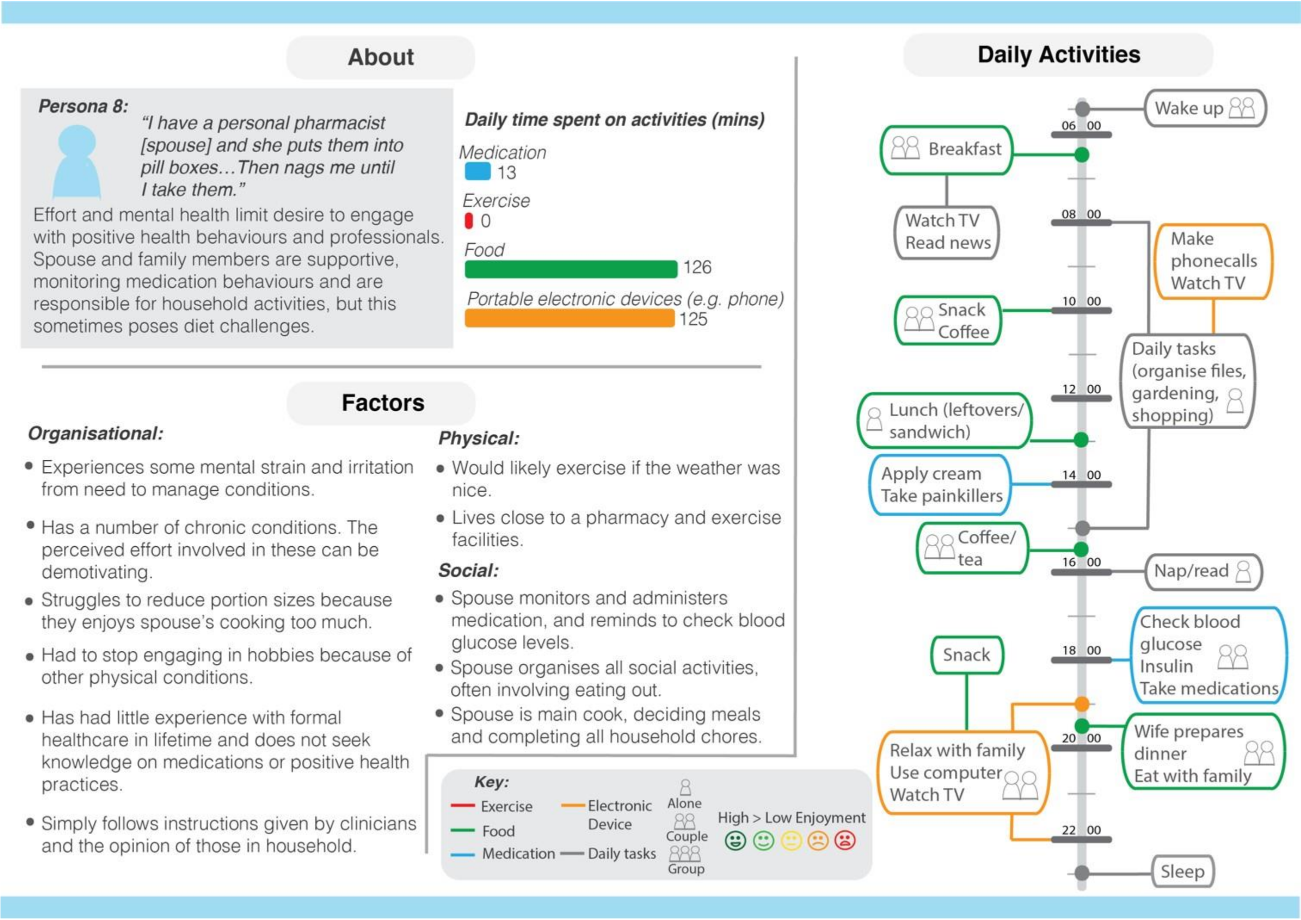

Supplement: Supplementary file 1 [file Data_Sheet_1.pdf]
