# Supplementary material for: Patient Work Personas of Type 2 Diabetes—A Data-Driven Approach to Persona Development and Validation
Source: Front Digit Health. 2022 Jun 23;4:838651. doi: 10.3389/fdgth.2022.838651 (PMC9260172; doi:10.3389/fdgth.2022.838651)
Supplement: Supplementary file 2 [file Data_Sheet_2.pdf]

Appendix 2 – Original participant interview questions

Introduction – purpose, consent, audio recording

- Purpose of today is to share our personas with the participant.
- Also, we want their feedback on whether the findings are accurate accounts of their daily routine, and how useful they feel these kinds of information would have for health informatic designers.
- Explain that the feedback session will take approximately one hour, where we will audio-record the session, with the participant’s permission. (Seek their permission to turn audio recording)

Think-Aloud Session on Accuracy and Usefulness for design

- Explain how the personas were made
- Explain that no identifying information has been presented. In addition, these personas are aggregated with other participants’ data.
- The purpose of these personas is to help others (e.g. clinicians, technology developers, health policy makers, other researchers) understand the intricate nature of type 2 diabetes management.

Persona

- Let participants read the persona
- Instruct that they should “talk through” what they are thinking as they are looking at the persona
- Conduct a ‘ranking exercise’ where the participant ranks the following components according to their perceived accuracy, and their perceived usefulness for designing interventions to help T2DM self-management
    - About (Quotes & Summary)
    - Bar graphs showing time spent on different activities
    - Timeline
    - Contextual factors
- Going down the list of ranked categories, ask participants:
    - Why did you rank this category here? If participants didn’t rank or ranked multiple components in the same way:
    - [Accuracy] Is this category an accurate description of your daily life?
    - [Usefulness] How useful do you think this category is for designers trying to design something digital to help you?

Ask these questions at the end

- What kind of information or features do you want to have in these personas?
- How would you want the information in these personas presented?
- Did you notice something about your daily life you did not notice before?
- Did we miss something that we should include?
- What are the elements about living with type 2 diabetes that you think it’s important for others to know? How can they be represented visually?
- Is there anything else that you would like to add?

Wrap-up

- Thank participants for their time and comments.
- They can keep the persona printout.

- Seek feedback from participant on ways to improve Feedback session, e.g. whether we should mail out participant persona before interview. If so, 1 week before the interview is - it sufficient time?
