# Supplementary material for: Patient Work Personas of Type 2 Diabetes—A Data-Driven Approach to Persona Development and Validation
Source: Front Digit Health. 2022 Jun 23;4:838651. doi: 10.3389/fdgth.2022.838651 (PMC9260172; doi:10.3389/fdgth.2022.838651)
Supplement: Supplementary file 3 [file Data_Sheet_3.pdf]

Appendix 3 – Online validation survey

Part 1: Informed consent and participant eligibility criteria.

Information sheet

Title of Study: Understanding and Designing for Contextual Elements in Type 2 Diabetes Self-Management

Department: UCLIC

Researcher(s): Natasha Galliford (natasha.galliford.14@ucl.ac.uk)

Principal Researchers: Ann Blandford (a.blandford@ucl.ac.uk), Annie Lau (annie.lau@mq.edu.au)

1. Invitation Paragraph

You are being invited to take part in my MSc research project. Before you decide it is important for you to understand why this study is being done and what participation will involve. Please take time to read the following information carefully and discuss it with others if you wish. Ask us if there is anything that is not clear or if you would like more information. Take time to decide whether or not you wish to take part. Thank you for reading this.

2. What is the project's purpose?

The aim of this project is to better understand people's needs and practises when managing their health, in particular the self-management of type 2 diabetes including diet, exercise, medication and physiological monitoring (e.g. blood glucose) management. This has a view to propose design suggestions for future digital interventions to aid in self-management practice, with a particular focus on contextual support (e.g. social, organisational and physical barriers to managing your diabetes).

3. Why have I been chosen?

You have been invited to participate because you are:

- Living with Type 2 Diabetes
- Aged 18 or over
- Able to communicate effectively in English and do not consider yourself to be a vulnerable adult.
- Able to give informed consent.

I am aiming to recruit up to 100 participants in total to participate in this study.

4. Do I have to take part?

It is up to you to decide whether or not to take part. If you do decide to take part you will be given this information sheet to keep and be asked to sign a consent form. You can withdraw at any time without giving a reason. If you decide to withdraw you will be asked what you wish to happen to the data you have provided up that point.

5. What will happen to me if I take part?

This study will involve an online questionnaire, taking approximately 10 minutes to complete.

You will be asked to provide demographic information, your behaviours when managing Type 2 Diabetes (e.g. exercise, diet and blood glucose monitoring) and your experiences of contextual factors (e.g. social influences and personal routines).

You will then be asked to read a series of personas that depict patients with Type 2 Diabetes and a comorbid condition. Personas are fictitious characters that depict individuals living with Type 2 diabetes, where information on their personal routines, how they spend their time, and what influences their actions and decisions will be presented. You will be asked your opinions on the personas shown to you, including the extent to which they are representative of your personal experience in managing Type 2 Diabetes.

Finally, you will be asked about your use of, and experiences with, digital technologies that aim to promote healthy behaviours for Type 2 diabetes (e.g. exercise, diet management). For instance, mobile apps or devices that aim to support healthy eating or fitness.

In total, your time in this study should take approximately 10 minutes.

6. What are the possible disadvantages and risks of taking part?

No disadvantages or risks of taking part have been identified. In the unlikely event that participating causes you any distress, you are free to withdraw, to discuss concerns with the researcher or the Principal Investigators.

7. What are the possible benefits of taking part?

While there are no immediate benefits to you from taking part, we hope that you will find the study interesting and that it will help you to reflect on how you manage, perceive or approach Type 2 diabetes self-management.

8. What if something goes wrong?

If you have any concerns with the conduct of this study, please raise them in the first instance with Professor Ann Blandford (a.blandford@ucl.ac.uk) or Dr Annie Lau (annie.lau@mq.edu.au). If your concerns are not addressed to your satisfaction then you may contact the Chair of the UCL Research Ethics Committee – ethics@ucl.ac.uk

9. Will my taking part in this project be kept confidential?

All the information that we collect about you during the course of the research will be kept confidential, subject to legal constraints and professional guidelines. You will not be identifiable in any ensuing reports, presentations or publications.

10. What will happen to the results of the research project?

This study is for my MSc project, and the findings will be reported in my dissertation. If you would like to receive a copy of that, let me know and I will send it to you in August. Depending on the findings, my supervisor and I may also publish the results in a journal or conference paper. Anonymised data will be stored securely for five years, and may be reviewed in subsequent studies that have a related focus.

11. Local Data Protection Privacy Notice

The controller for this project will be University College London (UCL). The UCL Data Protection Officer provides oversight of UCL activities involving the processing of personal data, and can be contacted at data-protection@ucl.ac.uk

The only personal information retained will be a copy of your informed consent and your chosen contact details if you wish to be informed of the outcome of this study. These will be held securely and separately from the anonymised data that you provide for the study.

Further information on how UCL uses participant information can be found at https://www.ucl.ac.uk/legal-services/privacy/ucl-general-research-participant-privacy-notice

The lawful basis that would be used to process your personal data will be performance of a task in the public interest.

12. Contact for further information

Contact details for me and my supervisor are provided at the top of this sheet; feel free to contact either of us if you have queries or concerns.

Thank you for reading this information sheet and for considering taking part in this study.

Consent Form:

1. I confirm that I have read and understood the Information Sheet for the above study. I have had an opportunity to consider the information and what will be expected of me and to ask questions which have been answered to my satisfaction. I agree to take part in an online questionnaire.
2. I understand that data will be anonymised and that it will not be possible to link my personal data (consent, contact details) with the study data. I understand that according to data protection legislation, 'public task' will be the lawful basis for processing.
3. I understand that all personal information will remain confidential and that all efforts will be made to ensure I cannot be identified. Data gathered in this study will be stored anonymously and securely. It will not be possible to identify me in any publications.
4. I understand that my information may be subject to review by responsible individuals from the University for monitoring and audit purposes.
5. I understand the direct/indirect benefits of participating.
6. I understand that I will not benefit financially from this study or from any possible outcome it may result in in the future.
7. I understand that I will be compensated for the portion of time spent in the study if I choose to withdraw.
8. I agree that my anonymised research data may be used by others for future research. [No one will be able to identify you when this data is shared.]
9. I understand that the information I have submitted will be published as a report and I wish to receive a copy of it. Yes/No

10. I confirm that I understand the inclusion criteria as detailed in the Information Sheet and explained to me by the researcher and that I fall under the inclusion criteria.

11. I am aware of who I should contact if I wish to lodge a complaint.

I confirm that I agree to points 1-11 outlined above and wish to take part in this survey.

I CONSENT to take part in this study

I DO NOT CONSENT to take part in this study

You can withdraw from the study at any point by exiting the browser.
Should you wish to withdraw your responses at a later date please contact natasha.galliford.14@ucl.ac.uk by the 31st July.

Please press continue to begin the study.

Are you aged 18 or over?

Yes

No

Are you fluent in English?

Yes

No

Have you been diagnosed with Type 2 Diabetes?

Yes

No

Part 2: T2DM management practices

For how many YEARS have you been diagnosed with Type 2 Diabetes? Please round to nearest year.

What type of medications do you take to control your diabetes? Select all that apply.

- Insulin
- Oral medication (e.g. tablets)
- Other

How many times a day, on average, do you measure your blood glucose levels?

- I do not test my own blood glucose levels
- Less than once per day
- Once per day
- Twice per day
- Three times per day
- More than three times per day

(If DID NOT select "I do not test my own blood glucose levels):

How do you use the results of your blood glucose tests? Select all that apply.

- To check/alter insulin
- To check/alter tablets
- To inform what I eat
- To inform how much exercise I do
- To help my diabetes care team alter my medication/treatment
- Other (please specify)

How would you rate your engagement in physical activity? (Note: an example of high physical activity could include engaging in regular exercise and/or monitoring of distances, time, calories etc.)

Very Low	Moderately Low	Somewhat Low	Somewhat High	Moderately High	Very High

How would you rate your control of your diet? (Note: an example of high diet control could include monitoring diet and/or restricting food groups, portion sizes or fast foods.)

Very Low	Moderately Low	Somewhat Low	Somewhat High	Moderately High	Very High

Which of the following factors have a POSITIVE impact on your lifestyle managing Type 2 Diabetes? Check all that apply

Flexible daily schedule (e.g. work from home)

Independence

Support from friends/family at maintaining healthy behaviours

Another household/family member prepared meals

Being the main person to prepare meals for self or others in the household

Able to form routines

Motivation

Knowledge gained from professionals (e.g. clinicians, dieticians, exercise specialists)

Social activities (e.g. hobbies, events)

Other competing priorities (e.g. raising children, carer responsibility)

Other(s) (please specify)

None

Which of the following factors have a NEGATIVE impact on your lifestyle managing Type 2 Diabetes?
Check all that apply

Inflexible daily schedule (e.g. long working hours)

Lack independence

Lack suppo11fr om friends/family at maintaining healthy behaviours

Another household/family member prepares meals

Being the main person to prepare meals for self or others in the household

Unable to form routines

Lack motivation

Lack/poor knowledge gained from professionals (e.g. clinicians, dieticians, exercise specialists)

Social activities (e.g. hobbies, events)

Other competing priorities (e.g. raising children, carer responsibility)

Other(s) (please specify)

None

Part 3: Persona evaluation

Here, you will be shown 2 personas that have been developed based on individuals living with Type 2 diabetes.

Personas are fictitious characters. Each persona contains information about individuals living with Type 2 diabetes, how they spend their time, and what are the factors that influence their actions and decisions.

Please read each persona and provide a rating of how similar it is of your life in managing type 2 diabetes.

Image of persona and its description

On the following scale, please select how SIMILAR (or different), you feel this persona is of your life in managing Type 2 Diabetes:

Very Different	Moderately Different	Somewhat Different	Neutral	Somewhat Similar	Moderately Similar	Very Similar

Which sections of this persona contain information that are SIMILAR to your experiences in managing Type 2 Diabetes. Select all that apply.

- Quotes and/or summary
- Graphs showing time spent on medication, food, exercise and electronics
- Timeline showing daily activities (e.g. order of tasks, enjoyment, complete things alone or as a group)
- Physical factors (e.g. tracking behaviours, distance to facilities, weather, nature of work)
- Social factors (e.g. level of support from others, social activities, influence of family routines, independence)
- Organisational factors (e.g. routines, life disruptions/priorities, experiences with healthcare professionals)
- Other(s) (please specify)
- None

Which sections of this persona contain information that are DIFFERENT to your experiences in managing Type 2 Diabetes. Select all that apply.

- Quotes and/or summary
- Graphs showing time spent on medication, food, exercise and electronics
- Timeline showing daily activities (e.g. order of tasks, enjoyment, complete things alone or as a group)
- Physical factors (e.g. tracking behaviours, distance to facilities, weather, nature of work)
- Social factors (e.g. level of support from others, social activities, influence of family routines, independence)
- Organisational factors (e.g. routines, life disruptions/priorities, experiences with healthcare professionals)
- Other(s) (please specify)
- None

Image of persona and its description

Repeat above questions again.

Part 4: T2DM changes and digital interventions

What areas of your lifestyle would you like to CHANGE for your Type 2 Diabetes? Select all that apply.

- Amount of physical activity
- Tracking of physical activity
- Forming an exercise routine
- Restricting food groups OR portion sizes
- Diet tracking
- Sugar intake
- Time spent eating out
- Medication organisation
- Increase blood glucose monitoring
- Motivation
- Independence
- Other(s) (please specify)
- None

In your opinion , what would be an ideal technology or dig it al resource to help you make these change{s)?

Activity tracker (e.g. Fitbit .)

Smartwatch

Other wearable technology {e.g. smart trainers)

Health-related mobile application (e.. g. calorie tracker) (p lease specify)

____

Mobile application (please specify)

____

Social media (e.g. Facebook, Instagram) (please specify)

____

Smart speaker (e.g. Alexa, Google home)

Smartphone voice assist ant (e.g . Siri

Smart scales

Virtual or augmented reality

Continuous blood glucose sensors (e.g.. IFreeStyle Libre)

Automatic/continuous insulin pumps

Other(s) (plea se specify)

____

None: Technology could not help me make these change(s)

Part 5: Demographic information and digital literacy

What is your age?

- Under 18
- 18 - 24
- 25 - 34
- 35 - 44
- 45 - 54
- 55 - 64
- 65 - 74
- 75 - 84
- 85 or older

What is your gender?

- Male
- Female
- Other (please specify)
- Prefer not to say

What is your current employment status?

Employed full-time

Employed part-time

Unemployed

Student

Retired

Self-employ ed

Unable to work

What is your marital status?

Single (never married)

Married

In a domestic partnership

Divorced

Widowed

Prefer not to say

Including you, how many people liv e in your household? (Include those who have been living/ staying here for more than 2 months}

2

3

4

5

6+

Rate how comfortable you are in using digital devices for your EVERYDAY LIFE:

Very Uncomfortable	Moderately Uncomfortable	Somewhat Uncomfortable	Not Applicable	Somewhat Comfortable	Moderately Comfortable	Very Comfortable

Rate how comfort able you are in using digital devices for your HEALTH:

Very Uncomfortable	Moderately Uncomfortable	Somewhat Uncomfortable	Not Applicable	Somewhat Comfortable	Moderately Comfortable	Very Comfortable
