# Supplementary material for: Patient Work Personas of Type 2 Diabetes—A Data-Driven Approach to Persona Development and Validation
Source: Front Digit Health. 2022 Jun 23;4:838651. doi: 10.3389/fdgth.2022.838651 (PMC9260172; doi:10.3389/fdgth.2022.838651)

Appendix 4 – Bar graphs for accuracy and usefulness form original participants (n=10)

Persona components ranked by perceived accuracy (1= most accurate out of the four components, 4=least accurate)

A

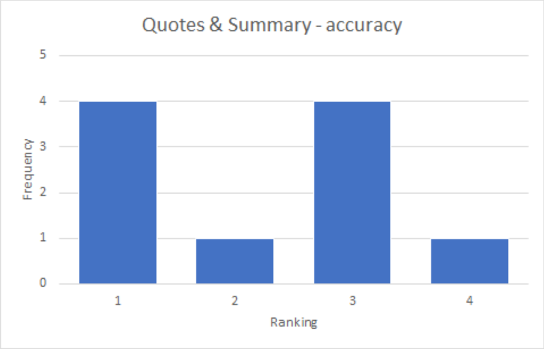

B

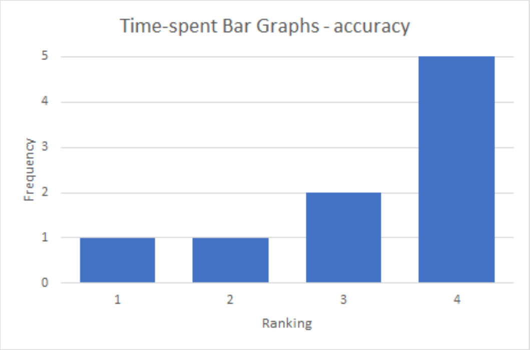

C

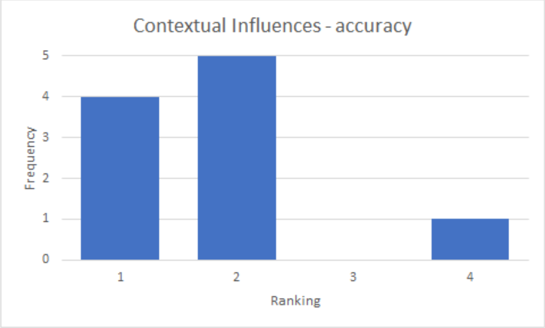

D

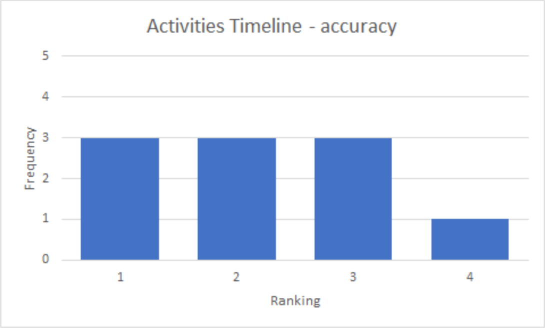

Persona components ranked by perceived usefulness (1= most accurate out of the four components, 4=least accurate)

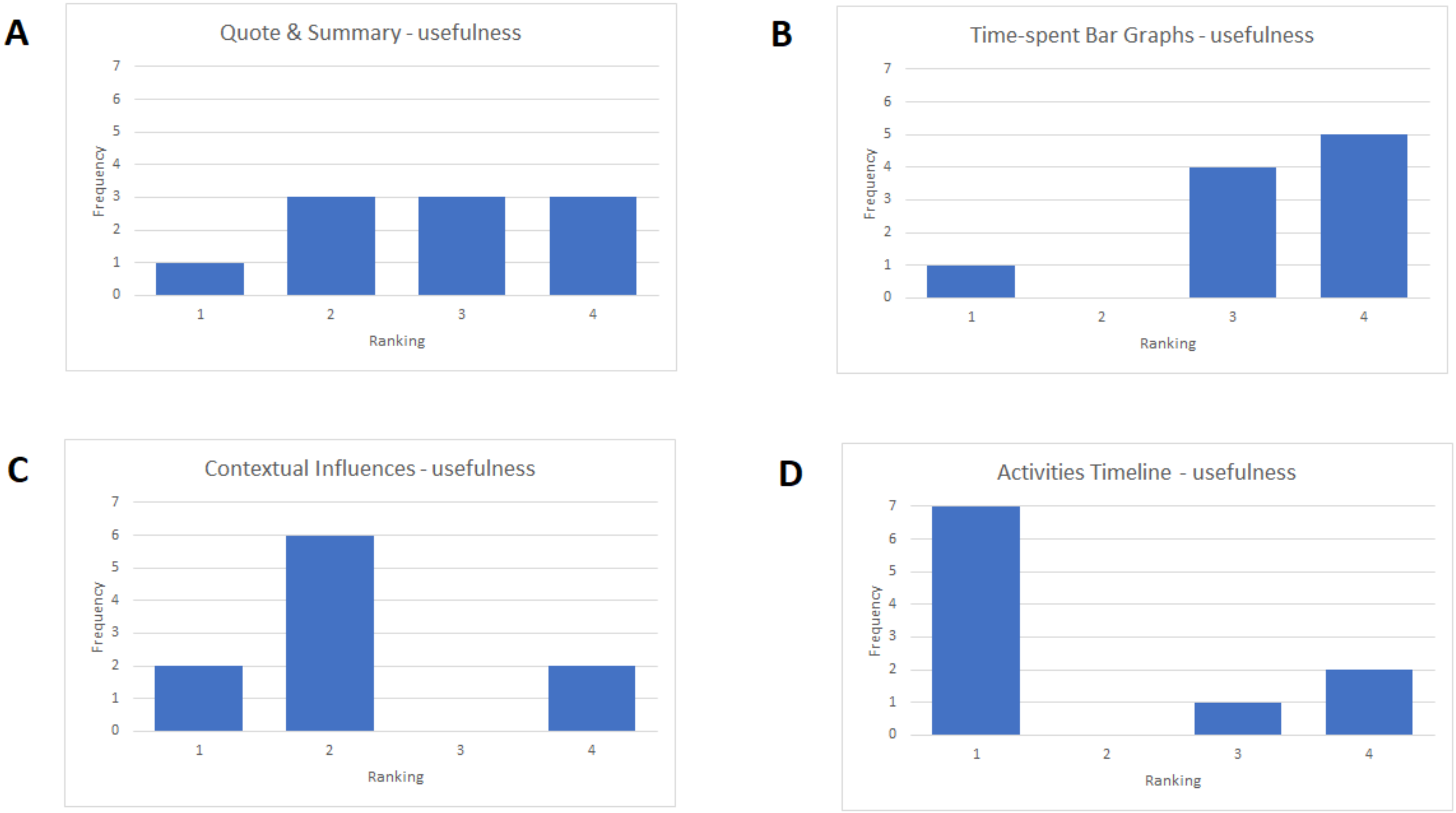

Supplement: Supplementary file 4 [file Data_Sheet_4.pdf]
